# Plasma Proteome Alterations of Laying Hens Subjected to Heat Stress and Fed a Diet Supplemented with Pequi Oil (*Caryocar brasiliense Camb.*): New Insights in the Identification of Heat Stress Biomarkers

**DOI:** 10.3390/biom14111424

**Published:** 2024-11-08

**Authors:** Joyce da Silva, Luane Andrade, Paola Rodrigues, Laís Cordeiro, Gabrieli Lima, Júlia Lopes, Elis Castillo, Renata Martins, Andrey Assunção, José Vieira, Marília Busalaf, Jiri Adamec, José Sartori, Pedro Padilha

**Affiliations:** 1 School of Veterinary Medicine and Animal Science, São Paulo State University (UNESP), Botucatu 18618-681, SP, Brazil; joyce.silva@unesp.br (J.d.S.); luane.andrade@unesp.br (L.A.); paola.damazio@unesp.br (P.R.); lais.cordeiro@unesp.br (L.C.); gabrieli.lima@unesp.br (G.L.); jl.lopes@unesp.br (J.L.); e.castillo@unesp.br (E.C.); renata.a.martins@unesp.br (R.M.); asa.assuncao@unesp.br (A.A.); jose.sartori@unesp.br (J.S.); 2 Institute of Biosciences, São Paulo State University (UNESP), Botucatu 18618-693, SP, Brazil; cavalcante.vieira@unesp.br; 3 Faculty of Dentistry of Bauru (FOB), University of São Paulo (USP), Bauru 17012-901, SP, Brazil; mbuzalaf@fob.usp.br; 4 School of Medicine, Louisiana State University Health Sciences Center (LSUHSC), New Orleans, LA 70112, USA; jadame@lsuhsc.edu

**Keywords:** antioxidant compounds, phytogenics, oxidative stress, poultry, shotgun–LC-MS/MS

## Abstract

Heat stress can disrupt the balance between the heat poultry release into the environment and the heat they generate. Pequi oil has antioxidant properties, which may mitigate the heat stress effects. This study aimed to investigate the response of laying hens to pequi oil supplementation under heat stress using a proteomic approach. A total of 96 Lohmann White laying hens with 26 weeks old were housed in a completely randomized design with a 2 × 2 factorial arrangement. They were housed in two climate chambers, thermal comfort temperature ± 24.04 °C with the relative humidity ± 66.35 and heat stress (HS) ± 31.26 °C with the relative humidity ± 60.62. They were fed two diets: a control diet (CON), basal diet (BD) without additives, and with Pequi oil (PO), BD + 0.6% PO. After 84 days, plasma samples were analyzed using Shotgun and LC-MS/MS. Proteins related to anti-inflammation, transport, and the immune system were differentially expressed in hens fed PO and CON under heat stress compared to those in thermoneutral environments. This helps protect against oxidative stress and may support the body’s ability to manage heat-induced damage, stabilizing protein expression under stress conditions. The ovotransferrin proteins, fibrinogen isoforms, apolipoprotein A-I, Proteasome activator subunit 4, Transthyretin, and the enzyme serine Peptidase Inhibitor_Kazal Type 5, which presented Upregulated (Up) equal to 1, present characteristics that may be crucial for enhancing the adaptive responses of hens to thermal stress, thereby increasing their tolerance and minimizing the negative effects of heat on egg production. The data presented in this manuscript provides new insights into the plasma proteome alterations of laying hens fed a diet supplemented with pequi oil during heat stress challenges.

## 1. Introduction

Intensive poultry production subjects birds to numerous stressors and environmental issues. Specifically, the increasing frequency and severity of heat waves is concerning, leading to heat stress (HS), a condition in which there is an imbalance in poultry between the heat they release to the environment and the heat they generate. This significantly impacts poultry growth and productivity, becoming a major challenge for the poultry industry [1]. Birds initiate an integrated response to maintain homeostasis through the interplay between the central nervous, endocrine, and immune systems [2]. In laying hens, HS leads to economic losses due to reduced feed intake, resulting in insufficient nutrient intake and lower egg weight, as well as reduced shell mineralization and general welfare [3,4,5].

These stressors can cumulatively affect poultry behavior and physiology, affect the immune response, and induce immunologically mediated or immune stress [2,6]. Immune stress is detrimental to birds and can be mitigated by improving their environment [7]. Studies have shown that stress dysregulates the immune response by increasing the release of inflammatory cytokines and stress hormones [8,9]. Acute phase proteins (APP) are a group of proteins primarily synthesized in hepatocytes and released into the bloodstream in response to challenges such as bacterial infection, inflammation, tissue injury, endotoxin exposure, and neoplasia [10,11]. Functions of APP include protease inhibitors, enzymes, transport proteins, coagulation proteins, and modulators of the immune response, which can also be elevated under conditions of chronic stress, contributing to prolonged inflammatory and immune responses.

Proteins are indispensable for many cellular functions, including metabolic activities, cell fate determination, and maintenance of overall body balance [12]. Proteomic analysis is a key technique for uncovering the molecular mechanisms of diseases and cellular metabolism and has gained widespread use in studying cellular processes and signaling pathways, significantly advancing our understanding of biology and medicine [7].

Pequi is a fruit with a yellowish-orange pulp rich in carotenoids, vitamins (A, C, and E), and minerals [13]. Its antioxidant properties inhibit the formation of free radicals, eliminate reactive oxygen species (ROS), break down peroxides, and reduce the local oxygen concentration [14]. These properties highlight the potential health benefits of pequi, which contains phenolic compounds that can donate electrons to free radicals, stabilize molecules, and prevent oxidation, stimulating the antioxidant system, attenuation of inflammatory responses, and reduction of oxidative stress in broilers under heat stress conditions, along with hepatoprotective effects and mitigation of negative impacts reported by Cruvinel et al. [15].

It has fatty acids in its composition, such as oleic acid in greater quantity, with a high content of carotenoids, including β-carotene, ζ-carotene, cryptoflavin, β-cryptoxanthin, antheraxanthin, zeaxanthin and mutatoxanthin, and phenolic compounds, such as ellagic acid, gallic acid, 4-hydroxy benzoic acid and p-coumaric acid [16,17]. The phenolic compounds present in the pequi fruit, mainly flavonoids, contribute to the antioxidant and anti-inflammatory properties of its oil. The fruit is considered an adaptogen, acting as a natural metabolic regulator that enhances the body’s ability to adapt to environmental factors and prevents damage caused by these factors, including those resulting from oxidative stress [18,19,20,21].

In the present study, this model was used to investigate how pequi oil influences protein expression in the blood plasma of laying hens under heat stress using a proteomic approach. The findings could enhance our understanding of how pequi oil affects hens’ physiology and their ability to cope with environmental stressors.

The experiment was conducted at São Paulo State University (UNESP), College of Veterinary Medicine and Animal Science (FMVZ), Botucatu, at Laboratory of Poultry Nutrition, approved by the Ethics Committee on the Use of Animals–CEUA (protocol n° 9 221/2022) of this institution, in accordance with animal experimentation and welfare animal. Birds were vaccinated for Marek’s disease, Avianpox, and Gumboro disease upon hatching. Until the 26th week of age, they were vaccinated against Gumboro disease, infectious bronchitis, Newcastle disease through the eye, Bouba in the wing membrane, Egg Drop Syndrome and Infectious Coryza oily via the intramuscular route.

## 2. Materials and Methods

### 2.1. Chicks, Housing, Management and Diets

Ninety-six Lohmann White laying hens (26-week-old) were distributed in a completely randomized design with a 2 × 2 factorial arrangement of treatments (two diets and two temperatures). They were housed in 24 galvanized wire cages, with 6 cages containing 4 birds (0.5 m height × 0.5 de length × 0.6 m width). Water was provided through nipple drinkers and trough feeders were positioned in front of the cages. The hens were allocated to two climate chambers (cyclic heat stress and thermoneutral) with two diets: control diet (CON), basal diet (BD) without additives, and with Pequi oil (PO), BD + 0.6% PO. All diets were formulated to meet the requirements of the hens with isocaloric and isonutritive attributes, based on corn and soybean meal according to the recommendations of [22] (Table 1). PO was added to the diet to replace soybean oil, considering the correction for the PO apparent metabolizable energy (AME) value of 7370 kcal/kg [15]. The birds were provided with water and food ad libitum.

**Figure 1:**
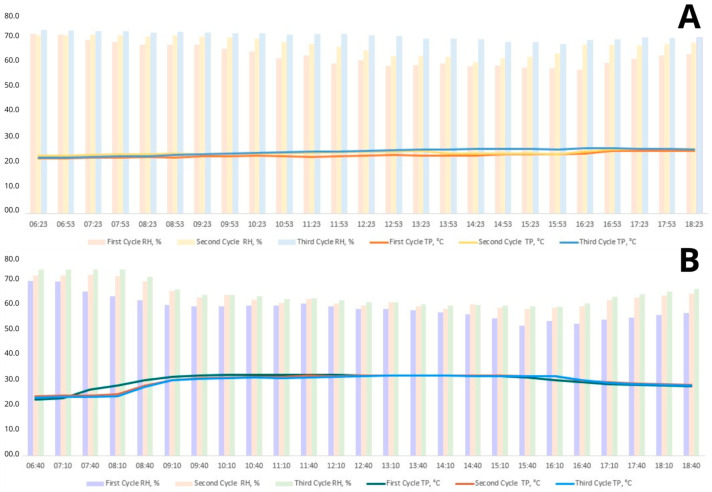
Temperature (TP) and Relative Humidity (RH) per week. First cycle (26, 27, 28, 29 wks), Second cycle (30, 31, 32, 33 wks), and Third cycle (34, 35, 36, 37 wks). (**A**)—Termoneutral; (**B**)—Heat stress.

The lighting program of 16 h daily (natural and artificial) was applied according to the management guidelines and recommendations of the lineage. The birds in the thermoneutral room, remained at a thermal comfort temperature ± 24.04 °C with the relative humidity ± 66.35. In the cyclic heat stress room, the birds were exposed to a temperature of heat stress (HS) ± 31.26 °C with the relative humidity ± 60.62 daily for 8 h (8:30 a.m. to 4:30 p.m.) (Figure 1). After this period, the temperature was lowered to match with the thermoneutral room for the remaining 84 days of the experiment. Air temperature and relative humidity were monitored daily using maximum and minimum rate sensors placed at the height of the birds. HOBO-type electronic sensors (Onset Data Loggers) were used for accurate measurements.

### 2.2. Proteomic Analysis

#### 2.2.1. Plasma Blood Samples Collection

At the end of the experimental period (84 days), blood samples were collected from one bird per experimental unit (six per treatment) and processed for plasma separation with an anticoagulant, which was used for proteomic analysis by Shotgun–LC-MS/MS. After collection, the samples were immediately frozen in liquid nitrogen and transported to the Laboratory of Bioanalytics and Metalloproteomics (LBM) at the Department of Chemistry and Biochemistry, IBB-UNESP Botucatu. Subsequently, the samples were stored at −80 °C until use in the molecular assays. Proteomic analysis was performed using a pool of plasma samples from the experimental (treatment) groups.

#### 2.2.2. Samples Preparation and Proteolytic Cleavage

The Shotgun–LC-MS/MS strategy was employed to obtain the proteomes of the plasma samples from the control and experimental groups. For this purpose, 100 µL of plasma from each sample pool were used and 200 µL of buffer containing 600 μg of protein, determined by the method of Bradford [23], was diluted in 50 mmol L^−1^ ammonium bicarbonate, and the mixtures were incubated at 37 °C for 30 min. Subsequently, 9 μL of 100 mmol L^−1^ dithiothreitol was added, and the mixtures were incubated at 37 °C for 40 min. Next, 6.3 μL of 300 mmol L^−1^ iodoacetamide (BioRad, Hercules, CA, USA) was added, and the mixtures were incubated for 30 min at room temperature in the dark. Then, protein digestion was conducted by adding 40 μL of trypsin (Thermo Fisher, Waltham, MA, USA) for 14 h at 37 °C. The following day, 10 μL of 5% (*v/v*) trifluoroacetic acid (Sigma-Aldrich, St. Louis, MO, USA) was added, and the mixtures were incubated for 90 min at 37 °C. After trypsin digestion, the extracts were centrifuged at 14,000 rpm at 6 °C for 30 min. Supernatants were collected and purified using C18 spin columns (Thermo Fisher Scientific, Waltham, MA, USA). The extracts were resuspended in 12 μL of 1 pmol·μL^−1^ ADH + 22.108 μL of 3% (*v/v*) acetonitrile (Sigma-Aldrich, St. Louis, MO, USA) and 3% (*v*/*v*) formic acid (Thermo Fisher, Waltham, MA, USA). After this treatment, the extracts were analyzed using the nanoAcquity UPLC-Xevo QTof MS system (Waters, Manchester, UK) [24].

### 2.3. Statistical Analysis

Protein Lynx Global Server (PLGS) software (version 2.5) was use for peptide identification, applying the Monte Carlo algorithm and identifying the peptides using the UniProt database [24]. The PLGS software was also used to generate normalized protein abundance counts (expression analyses). In the expression analyses, *p* < 0.05 was considered for negatively regulated proteins and 1 − *p* > 0.95 for positively regulated proteins [25]. After compiling the results, all sequences of differentially expressed proteins were submitted to functional analysis using STRING database software (Java 9 New Version) to identify the functional protein categories based on gene ontology annotations of biological processes and to identify protein-protein interactions [24,25].

## 3. Results

The mass spectrometry results were imported into the database, and 74 proteins were identified. The proteins identified in the blood plasma of laying hens in the PO and CON groups under heat stress and those in a thermoneutral room. Among the proteins identified in the PO and CON comparations, 58 were upregulated and 16 were downregulated. These were analyzed based on two criteria: *p* ≥ 0.1 to ≥0.5 was considered negative regulation and can be visualized by the blue color gradient, and *p* ≥ 0.95 to ≥1 was considered positive regulation and can be visualized by the green color gradient. These proteins were classified according to their research interests and are presented in Table 2. The raw data of mass spectrometry referents to protein identification are shown in Supplementary Material file.

**Figure 2:**
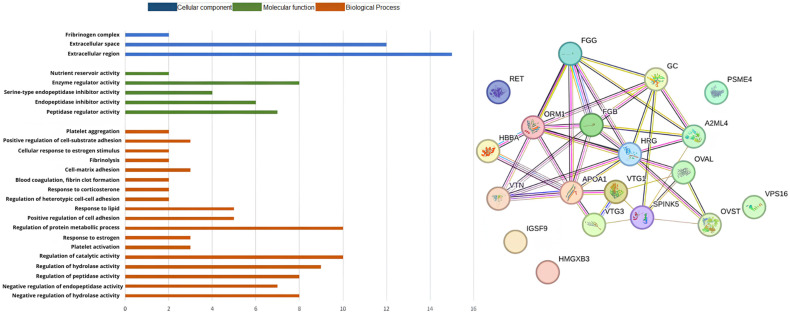
Classification and interaction of proteins in the CON^ST^ group characterized in the plasma proteome of laying hens using the STRING software.

**Figure 3:**
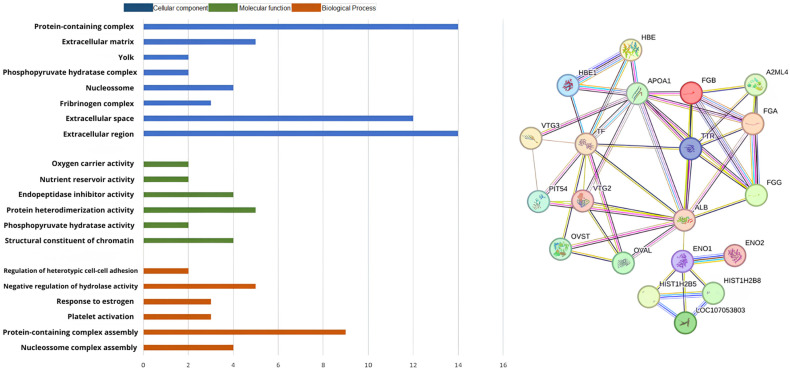
Classification and interaction of proteins in the PO^ST^ group characterized in the plasma proteome of laying hens using the STRING software.

The gene ontology analysis was used to classify the protein sequences into biological processes (BP), molecular functions (MF), and cellular components (CC). GO functional analysis showed that the proteins identified when the diet was CON^ST^ in the BP group were primarily involved in the regulation of catalytic activity (10), protein metabolic processes (10), hydrolase activity (9), negative regulation of hydrolase activity (8), peptidase activity (8), and endopeptidase activity (7). In the MF group, the genes were primarily involved in enzyme (8), peptidase (7), and endopeptidase inhibitor activities (6). In the CC group, they were primarily located in the extracellular (15) and space (12) regions, as shown in Figure 2. In the GO functional analysis of the proteins identified in the PO^ST^ group, those in the BP were primarily involved in protein-containing complex assembly (9), negative regulation of hydrolase activity (5), and nucleosome assembly (4). In the MF group, these genes were primarily involved in protein heterodimerization (5), structural constituents of chromatin (4), and endopeptidase inhibitor activity (4). In the CC, were primarily involved in the extracellular region (14), protein-containing complexes (14), and extracellular space (12), as shown in Figure 3.

**Figure 4:**
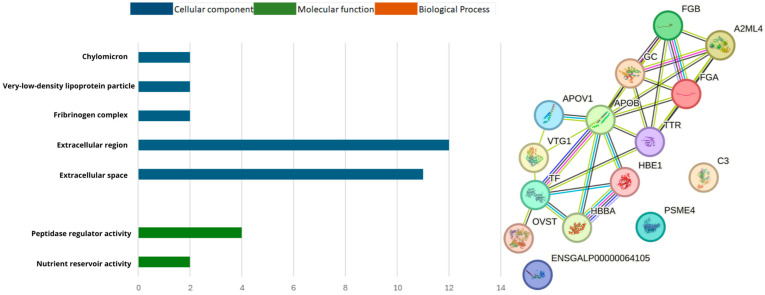
Classification and interaction of proteins in the C^S^ x P^S^ group characterized in the plasma proteome of laying hens using the STRING software.

**Figure 5:**
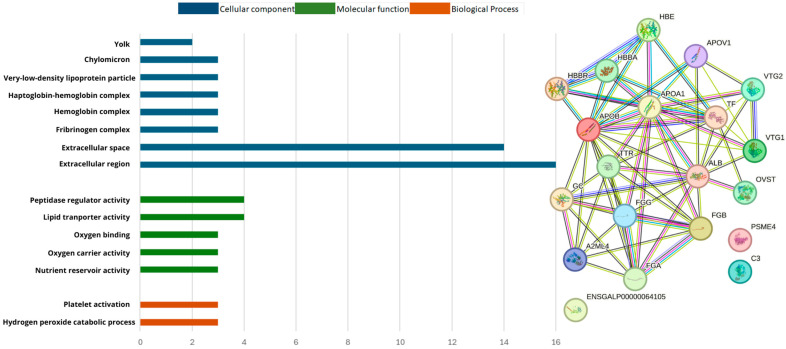
Classification and interaction of proteins in the C^T^ x P^T^ group characterized in the plasma proteome of laying hens using the STRING software.

In the MF group of C^S^ x P^S^ (Figure 4), the genes were primarily involved in nutrient reservoir activity (2) and peptidase regulator activity (4), in CC group, were primarily located in the extracellular space (11), extracellular region (12), fibrinogen complex (2), very-low-density lipoprotein particle (2) and chylomicron (2). The BP group in the GO functional analysis of the proteins identified in the C^T^ x P^T^ (Figure 5), were primarily involved in hydrogen peroxide catabolic process (3) and platelet activation (3). The MF were related of nutrient reservoir activity (3), oxygen carrier activity (3), oxygen binding (3), lipid transporter activity (4) and peptidase regulator activity (4), in CC group involved in extracellular region (16), extracellular space (14), fibrinogen complex (3), hemoglobin complex (3), haptoglobin-hemoglobin complex (3), very-low-density lipoprotein particle (3), chylomicron (3) and yolk (2).

**Figure 6:**
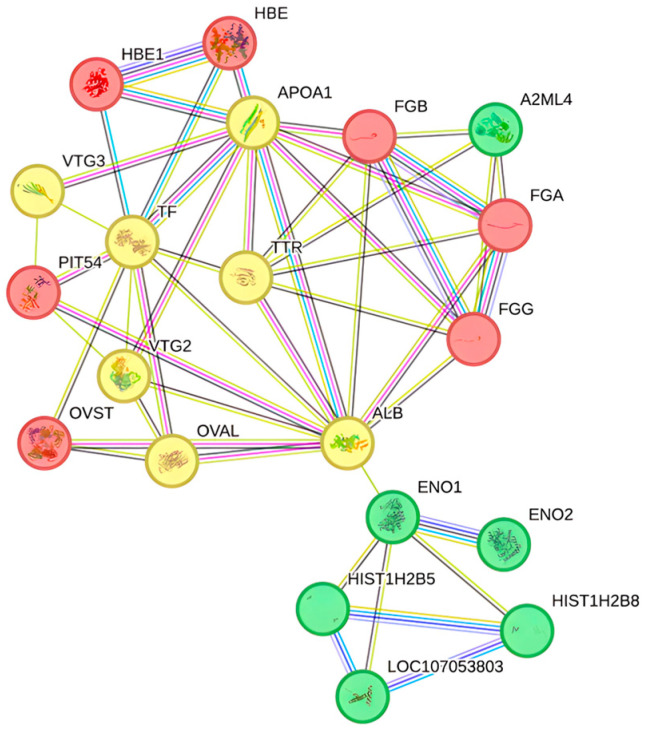
Cluster interactions of proteins that were regulated by the inclusion of PO^ST^ in the diet.

A protein–protein interaction network, differentially expressed in the plasma blood of laying hens supplemented with PO^ST^, was constructed using the String program and the Gallus gallus genome (Figure 6). The interactions revealed the proteins regulated by the inclusion of PO in the diet and how they collectively contributed to a shared function. The network was clustered into three groups: proteins involved in anti-inflammatory activity (red cluster), substance transport (yellow cluster), and immune system proteins (green cluster).

## 4. Discussion

### 4.1. Anti-Inflammatory Protein

The response to high temperatures is a critical adaptive stress mechanism for maintaining cellular homeostasis, including the induction of heat shock proteins (HSP) and other factors, such as the ubiquitin-proteasome system, which forms the basis of the heat shock response (HSR) in poultry [26,27]. Heat stress can regulate multiple metabolic proteins, and thermal denaturation begins with less-protected proteins, including receptors, epithelial transporters, and immune-related proteins [28]. These protective proteins can be categorized into two groups: heat shock proteins (HSPs) and acute-phase proteins (APPs) [29].

APPs vary in their response to stimuli; where the majority can increase 10- to 1000-fold within 1–2 days of stimulation, making them the primary responders in acute-phase reactions [11,30]. Moderate APPs show a lower concentration peak of around 5- to 10-fold, with their elevated levels persisting for more than 3 days [31,32]. In contrast, minor APPs showed only a minimal increase of up to 2-fold [33,34]. In poultry, APPs include Amyloid A, α1-acid glycoprotein, ovotransferrin, ceruloplasmin, fibrinogen, and fibronectin [11,31,35].

During inflammation, infection, or heat stress, the liver increases the production of Ovotransferrin (Tf) through the upregulation of proinflammatory cytokines as part of the acute-phase response (APR) [36]. In laying hens, Tf is produced under the regulation of estrogen and constitutes a significant component of egg white [37,38]. Its major physiological function is iron transport, and its antimicrobial properties are likely linked to its ability to sequester iron, an essential element for bacterial growth [39,40,41,42]. However, it was demonstrated that Tf is a redox-responsive protein, which plays a protective role in shielding the chick embryo from oxidants and may participate as a redox-dependent manner, potentially releasing free thiol groups, altering its structure to help neutralize ROS and protect the developing embryo by managing oxidative stress [42]. Upregulation of this protein was observed only with PO comparations (Table 2), indicating that its antioxidant properties may enhance the regulation of certain proteins and their protective abilities, making them more effective in health-related applications.

Fibrinogen is another glycoprotein that functions as an acute-phase protein with anti-inflammatory properties; however, it has only a minor influence on inflammatory reactions in poultry [43]. Hyperthermia causes direct thermal damage and compromises the integrity of the intestinal barrier [44]. This disruption facilitates the translocation of microbial toxins into the bloodstream, triggering a systemic inflammatory response, and potentially leading to organ dysfunction [45]. Heat stress triggers an APR, which can lead to increased production of this protein, as seen in hens with CON and PO, the upregulations of three different fibrinogen polypeptide chains: alpha (α), beta (β), and gamma (γ). Sudden death with clotted blood in the peritoneum is frequently seen in heat-stressed poultry because of high environmental temperatures [46].

Ke et al. [44] states that heatstroke is characterized by an elevated core temperature resulting from the dysfunction in the thermoregulatory processes and represents the most severe condition within the spectrum of heat-related illnesses, in their experiment with fibrinogen supplementation in rats with heatstroke significantly enhanced platelet aggregation, helping to improve platelet function, which causes impairment of platelet adhesion, spreading, and fibrin clot retraction in the rats with heatstroke was partially ameliorated by fibrinogen supplementation. Therefore, heat stress can affect fibrinogen levels and its role in hemostasis by boosting its production as a part of the APR. However, severe heat may also impair platelet function, disrupt clot stability, and alter overall hemostatic balance [44].

Haptoglobin (Hp) is a major APP in ruminants and is considered a moderate APP in humans, carnivores, horses, and rabbits. It is an α2-glycoprotein that belongs to the transport (metal-binding) conjugated proteins, which its primary function is to bind free hemoglobin (Hb), thereby protecting the organism from iron loss [47,48]. Georgieva [32] reported that Hp found in mammals and PIT 54 found in chickens (*Gallus gallus domesticus*) have similar functions, primarily involving binding to free hemoglobin to prevent iron loss and mitigate oxidative damage. PIT54 functionally converges with haptoglobin to maintain nitric oxide (NO) signaling, which is important for some physiological processes, including blood vessel dilation and immune responses [49]. Heat stress can lead to the breakdown of red blood cells, releasing free hemoglobin into the bloodstream by reacting with NO and reducing its availability [50,51]. Thus, the upregulated PIT54 found in hens with PO^ST^ supplementation can bind to free hemoglobins, reducing oxidative damage and protecting cells from harmful effects, helping to mitigate oxidative damage, maintaining NO function, and managing the adverse effects of heat stress in poultry.

Proteasome is a crucial protein complex in cells that breaks down and eliminates damaged or unnecessary proteins, particularly those that are oxidized, misfolded, or marked with a molecule called ubiquitin for degradation [52]. This process is essential for controlling protein levels and plays a role in regulating cell signaling, stress responses, and inflammation [53]. The downregulation of Proteasome activator subunit 4 (PSME4) in the CON^ST^ and its upregulation in the comparations with PO and CON in the diet, suggest that PSME4 may play a role in removing damaged, misfolded, or excess proteins, preventing their accumulation, which could otherwise disrupt cellular function under heat stress conditions.

### 4.2. Transport of Substances

The ability to bind free radicals is crucial for maintaining the balance between the oxidation and redox status in the body, which is important for normal physiological functions and thermoregulation [54]. Albumin is the most abundant circulating protein in the plasma and has significant antioxidant properties, such as a crucial role in protecting the cell against oxidative damage by trapping free radicals and binding to vulnerable lipids [55]. Polyunsaturated Fatty Acids (PUFAs) are a type of fat found that is particularly prone to oxidation due to their multiple double bonds, leading to the formation of harmful free radicals and oxidative stress, which can damage cells and tissues [56]. Hassan et al. [57] proposed that under heat stress, the body increases albumin levels to enhance this protective effect in an attempt to protect lipids from oxidation, helping to mitigate the damage caused by increased oxidative stress. Which can be seen on PO and C^T^ x P^T^ groups, where the upregulation of albumin may be a protective response to prevent lipid oxidation under stressful conditions.

Vitellogenin (VTG) and apolipoproteins (APOs) are synthesized in the liver under the influence of estrogen during egg laying in birds [58,59]. Vitellogenins are crucial because they provide the amino acids required for the synthesis of structural and non-structural proteins by the embryo and serve as immune effectors that protect the embryo from bacterial and viral infections [60,61]. High-Density Lipoprotein (HDL) and its primary protein component, apolipoprotein A1 (APOA1), help remove excess cholesterol from the peripheral cells and transport it to the liver [62]. Together, these proteins play critical roles in lipid metabolism, transport, and utilization in various organisms, ensuring that lipids are properly distributed throughout the body and facilitating energy production, cellular structure maintenance, and other essential biological functions [63]. Cheng et al. [64] suggested that the upregulation of APOA1 after heat stress in broiler chickens and layers may be a protective mechanism that involves exporting lipids into the bloodstream to prevent lipid peroxidation and lipotoxicity. This mechanism was also observed in the PO^ST^ and CON^ST^ groups. In the PO^ST^ group, by increasing vitellogenin 2 (VTG2) production, hens could better manage the negative impacts of heat stress on their reproductive systems, production, and quality, even under challenging conditions.

Transthyretin is a plasma protein primarily produced in the liver and choroid plexus of the brain. It functions as a transporter of thyroxine (T4), which can be converted into triiodothyronine (T3) by deiodinase enzymes, and T3 enhances the breakdown of fats into fatty acids and glycerol, increasing lipolysis, and raising body temperature [65,66]. In PO^ST^ and C^S^ x P^S^, upregulation of this protein typically results in increased metabolic activity.

### 4.3. Immune System

During heat stress, the immune system becomes immunosuppressed owing to the low expression of immunoglobulins and a reduction in the immune response [67]. The bird’s immune response can be enhanced through immunomodulation, achieved using targeted dietary supplementation and/or feed additives to alter immune function. This modulation aims to reduce inflammation, boost weakened responses, manage gut health, and play a significant role in maintaining immune function and health [68].

Upregulation of the IgM chain C region observed in the PO^ST^ and C^S^ x P^S^ group indicated an increased production of IgM antibodies. Gomes [69] suggested that stressors can lead to increased levels of immunoglobulins such as IgM, which is the first antibody produced in response to an infection and is crucial for the initial immune response. In addition, Ig-like domain-containing proteins play a role in both activating and inhibiting immune responses, and the upregulation of this protein in both groups could enhance regulatory pathways, thereby maintaining immune balance.

Serine proteinase inhibitors (SPIs) are crucial for regulating protease activity in many biological processes [70]. Kazal-type serine protease inhibitors (KSPIs) represent a well-established family of SPIs [71]. The serine Peptidase Inhibitor, Kazal Type 5 (SPINK5), a member of the KSPIs inhibitor family, is known for its role in inhibiting proteases and is particularly involved in regulating epithelial function and immune responses [72]. Proteases activate inflammatory pathways leading to ROS [73]. By inhibiting these proteases, KSPIs may help reduce inflammation and subsequent oxidative stress [71]. Downregulation of SPINK5, which was observed in the CON^ST^ group, can result in increased activity of serine proteases and may lead to uncontrolled inflammation and immune dysregulation, resulting in excessive protein breakdown and potential tissue damage.

### 4.4. Unique Proteins Express in PO^ST^ Group

Enolase, also known as phosphopyruvate hydratase, is an enzyme involved in the glycolytic pathway that catalyzes the conversion of 2-phosphoglycerate to phosphoenolpyruvate, which is crucial for cellular energy production [74]. The α-Enolase is involved in glycolysis, a pathway crucial for maintaining intracellular ATP levels in cardiomyocytes exposed to ischemic hypoxia, which is critical for cell survival [75]. Heat Shock Protein 70 (HSP70) protects cells against oxidative stress. It can interact with α-enolase to provide protection, particularly in cardiomyocytes, and this interaction suggests that α-enolase may work together with HSP70 to mitigate the effects of heat stress and oxidative damage [76]. Zeng et al. [74] found that α-enolase levels increased in Muscovy ducks under heat stress, suggesting that the α-enolase may play a role in managing the stress by potentially providing more energy or protecting cells. The findings suggest that α-enolase is multifunctional. Its role as an energy regulator by boosting energy availability during stress conditions, which may affect its activity and potentially contribute to pathological conditions in which oxidative stress is a factor.

The biological properties of von Willebrand factor (vWF) offer insights into the evolution of hemostasis and may shed light on the functions of vitellogenin [77,78]. vWF exhibits adhesive properties, including binding to platelets, collagen, and other proteins, and plays a crucial role in hemostasis by facilitating platelet adherence at sites of epithelial injury [79,80]. Additionally, vWF binds to factor VIII, with this binding site localized in the D domain, which is homologous to a region in vitellogenin, suggesting that the C-terminal portion of vitellogenin contributes to the evolution of the intrinsic coagulation [80,81]. The presence of vWF-like domains in vitellogenin in amphibians and birds highlights the conservation of these proteins across species. An intriguing potential function of this vitellogenin domain, reflecting the adhesive properties of von Willebrand factor, is its role in binding to the oocyte membrane receptor, which facilitates the uptake of vitellogenin into oocytes [82,83]. Inflammation increases during oxidative stress, potentially enhancing the activation of coagulation pathways and increasing von Willebrand factor (vWF) activity. Vitellogenin provides nutrients, and potentially influences oxidative stress. Both proteins are involved in key physiological processes, and their roles in oxidative stress may highlight their importance in managing cellular responses to damage. Understanding these interactions can provide insights into how they influence oxidative stress responses.

Modular protein domains encoded by specific amino acid sequences play a vital role in mediating protein-protein interactions, which are crucial for the regulation of numerous cellular processes, allowing proteins to form complexes, transmit signals, and perform their functions efficiently [84]. Understanding how these domains function is essential for the precise regulation of cellular functions and coordination of biological activities. Immunoglobulin V-set domain-containing proteins are part of a large family of proteins that includes domains structurally similar to the variable region (V-set) of immunoglobulins (antibodies) [85]. These domains are critical for the immune system and play many roles in immune responses and cell signaling [86].

Activated immune cells express new surface molecules containing immunoglobulin folds [87,88,89,90]. These molecules are crucial for cell recognition, adhesion, and migration, which are vital for T cell development and immune responses [91]. In addition to adhesion events influencing T cell stimulation, the activation state of T cells can also affect the differential expression of adhesion molecules. Thymocyte adhesion molecule expression is affected not only by immediate T cell stimulation, but also by the differentiated state of T cells. This regulation may determine the ability of the cell to transition rapidly between nonadherent and adherent states [92]. T-cell Receptors (TCRs) of the V-set domains are located in the variable regions of the α and β chains (or γ and δ chains in some T cells), which recognize and bind to antigenic peptides presented by major histocompatibility complex (MHC) molecules on the surface of antigen-presenting cell [83]. This modulation suggests that these molecules are essential for the differentiated functions of the cell, and by modulating these critical processes, they may help maintain cellular homeostasis and protect cells against the damaging effects of oxidative stress. Research into the specific functions and interactions of these proteins provides valuable insights into their potential therapeutic applications in diseases associated with oxidative stress, as shown in Table 3.

## 5. Conclusions

Proteomic analysis of blood plasma from laying hens fed with 0.6% pequi oil under heat stress confirmed the potential of pequi oil as a positive modulator of the immune system. This characteristic may be essential for enhancing the responses and adaptive mechanisms to thermal stress, thus increasing the tolerance of hens and minimizing the negative effects of heat on egg production.

## Figures and Tables

**Table 1:** Experimental diets: Ingredients and calculated chemical compositions.

Ingredients (%)	CON	PO 0.6%
**Corn**	58.393	58.393
**Soybean meal**	28.900	28.900
**Soybean oil**	2.000	1.497
**PO**	0.00	0.60
**Fine limestone**	1.600	1.600
**Coarse limestone**	5.400	5.400
**Dicalcium phosphate**	0.450	0.450
**Salt**	0.080	0.080
**DL-Methionine (99%)**	0.080	0.080
**Layer Premix**	3.000	3.000
**Inerte–Caulim**	0.097	0.000
**Total (Kg)**	100	100
**Apparent metabolizable energy (Kcal/Kg)**	2833	2833
**Crude protein**	17.50	17.50
**Calcium**	3.69	3.69
**Available phosphorus**	0.42	0.42
**Sodium**	0.17	0.17
**Lysine digestible**	0.84	0.84
**Methionine digestible**	0.39	0.39
**Met + Cys digestible**	0.63	0.63
**Tryptophan digestible**	0.20	0.20
**Threonine digestible**	0.59	0.59

PO: pequi oil; CON: control diet; Egg production premix (guaranteed levels per kg of product): Folic Acid (min): 15 mg/kg, Pantothenic Acid (min): 350 mg/kg, Biotin (min): 1.5 mg/kg, Calcium (min): 250 g/kg, Calcium (max): 270 g/kg, Copper: 225 mg/kg, Choline (min): 6.000 mg/kg, Iron: 1500 mg/kg, Phytase: 16.67 ftu/g, Fluorine: 460 mg/kg, Phosphorus: 49 g/kg, Iodine (min): 20 mg/kg, Manganese (min): 2.800 mg/kg, Methionine: 23.43 g/kg, Selenium: 6 mg/kg, Sodium (min): 45 g/kg, Niacin (min): 800 mg/kg, Vitamin A (min): 233.000 IU/kg, Vitamin B1 (min): 35 mg/kg, Vitamin B12 (min): 350 mcg/kg, Vitamin B2 (min): 100 mg/kg, Vitamin B6 (min): 100 mg/kg, Vitamin D3 (min): 80.000 IU/kg, Vitamin E (min): 333.333 IU/kg, Vitamin K3 (min): 60 mg/kg, Zinc (min): 2.000 mg/kg.

**Table 2:** Upregulated (Up) and downregulated (Down) proteins in the groups supplemented with 0.6% PO compared the control groups without PO supplementation under cyclic heat stress and thermoneutral conditions.

Accession	Description	Gene	^1^CON^ST^	^2^PO^ST^	^3^C^S^ x P^S^	^4^C^T^ x P^T^	
P19121	Albumin	*ALB*		1		1	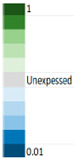
A0A8V0ZTN4	Alpha-2-Macroglobulin	*A2M*	0.98	1	1	0.01	
P08250	Apolipoprotein A-I	*APOA1*	1	1		0.03	
A0A8V1ACX8	A2ML1 protein	*A2ML4*			1	1	
Q197X2	Apolipoprotein B	*APOB*			0.02		
A0A8V0XGW5	Apovitellenin-1	*APOV1*			0.04	1	
Q7LZ77	Apolipoprotein B-100 (Fragment)	*-*				1	
A0A8V1AAP1	Alpha-2-macroglobulin-like protein 1	*A2ML4*				1	
Q90864	Beta-H globin	*-*		1	1		
Q90633	Complement C3	*-*			1	0.99	
A6N9E0	Complement component 3d (Fragment)	*C3d*			1		
P14448	Fibrinogen alpha chain	*FGA*		1	1	0.03	
A0A8V0YX35	Fibrinogen beta chain	*FGB*	0.98	1	1	0.01	
O93568	Fibrinogen gamma chain	*-*	1	1		0.04	
A0A8V0XM21	Hemoglobin subunit epsilon 1	*HBBA*		1	1	1	
P02128	Hemoglobin subunit epsilon	*HBE*	0.97	0.03		0.98	
P02112	Hemoglobin subunit beta	*HBB*	0.98			0.99	
P02127	Hemoglobin subunit rho	*-*				0.98	
A0A8V0X997	Hemopexin	*-*			1	1	
P01875	Ig mu chain C region	*-*		1	0.98		
A0A8V0XLK0	Ig-like domain-containing protein	*-*	1	1	1	0.04	
A0A8V0ZVD7	Immunoglobulin lambda-like polypeptide 1	*IGLL1*			1	1	
A0A8V0XA69	Immunoglobulin V-set domain-containing protein	*-*			1		
P20763	Ig lambda chain C region	*-*			0.01		
Q4ADJ7	Ovotransferrin	*TF*		1	1	0.98	
A0A8V0XEL0	PIT54 protein	*PIT54*		1			
A0A8V0Z5W0	Proteasome activator subunit 4	*PSME4*	0.01		0.99	1	
A0A8V0YI39	Serine peptidase inhibitor_ Kazal type 5	*SPINK5*	0.04				
P27731	Transthyretin	*TTR*		1	1	0.02	
Q6BCB8	Vitellogenin (Fragment)	*-*		1		1	
A0A8V0Z932	Vitellogenin 2	*VTG2*		1		1	
Q9W6F5	Vitamin D-binding protein	*VTDB*	1		1	0.02	
A0A8V0ZKX0	Vitellogenin 1	*VTG1*	1		0.99	0.01	

^1^CON stress and thermoneutral (CON^ST^); ^2^PO stress and thermoneutral (PO^ST^); ^3^CON stress and PO stress (C^S^ x P^S^); ^4^CON thermoneutral and PO thermoneutral (C^T^ x P^T^).

**Table 3:** Unique proteins express in PO^ST^ group.

Accession	Description	Gene	Score
**P51913**	Alpha-enolase	*ENO1*	81.44
**O57391**	Gamma-enolase	*ENO2*	81.44
**P0C1H3**	Histone H2B 1/2/3/4/6	*H2B-I*, *H2B-II*, *H2B-III*, *H2B-IV*, *H2B-VI*	345.93
**P0C1H4**	Histone H2B 5	*H2B-V*	345.93
**P0C1H5**	Histone H2B 7	*H2B-VII*	86.92
**Q9PSW9**	Histone H2B 8	*H2B-VIII*	345.93
**A0A8V0YNS6**	Histone H2B	*HIST1H2B5*	345.93
**A0A8V0XA69**	Immunoglobulin V-set domain-containing protein	*-*	523.52
**A0A8V0Z0S7**	phosphopyruvate hydratase	*ENO*	81.44
**A2N890**	VH1 protein (Fragment)	*VH1*	523.52
**A0A8V0ZG90**	Vitellogenin 3	*VTG3*	24.74
**A0A8V0X6K1**	VWFD domain-containing protein	*LOC429249*	22.72
**P01012**	Ovalbumin	*SERPINB14*	244.84

## Data Availability

Data contained within the article.

## Supplementary Materials

The following supporting information can be downloaded at: https://www.mdpi.com/article/10.3390/biom14111424/s1, Table S1: Raw data of the proteins identified in blood plasma proteome of laying hens supplemented with pequi oil (*caryocar brasiliense camb.*) under cyclic heat stress by Shotgun-liquid chromatography-tandem mass spectrometry (Shotgun-LC-MS/MS).
